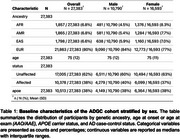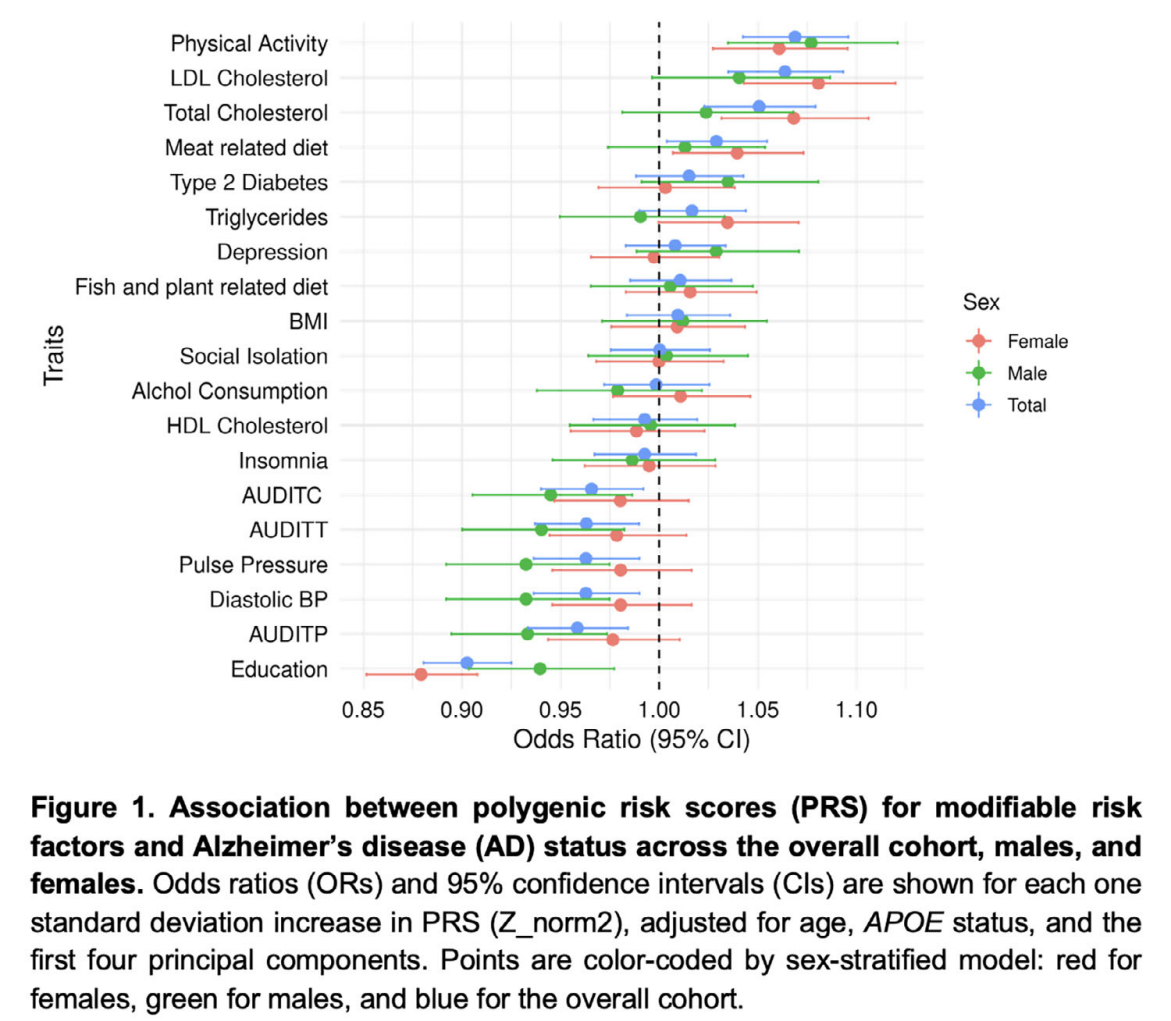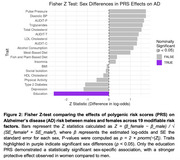# Polygenic Risk Scores for modifiable risk factors show sex‐specific associations in Alzheimer’s Disease

**DOI:** 10.1002/alz70861_108479

**Published:** 2025-12-23

**Authors:** Rakshya U Sharma, Meri Okorie, Nia Spadoni, Aadrita Chatterjee, Ana I Boeriu, Paulina Tolosa Tort, Kristine Yaffe, Shea J Andrews, Alzheimer's Diseases Genetics Consortium

**Affiliations:** ^1^ University of California, San Francisco, San Francisco, CA USA; ^2^ Department of Psychiatry and Behavioral Sciences, University of California ‐ San Francisco, San Francisco, CA USA; ^3^ Department of Psychiatry, University of California San Francisco, San Francisco, CA USA

## Abstract

**Background:**

Alzheimer’s disease (AD) is a major public health crisis, with two‐thirds of diagnoses occurring in women. The 2024 Lancet Commission of dementia prevention attributed 45% of AD cases to modifiable risk factors such as physical activity, cholesterol levels, and education; which also have sex specific prevalences. Our study aims to understand whether genetic liability for these risk factors show sex‐specific associations with AD.

**Method:**

We constructed 19 PRS in the Alzheimer’s Disease Genetics Consortium (ADGC; N=27,383; 60.6% female; mean age=75 [SD=12]; 37.8% cases; 80% European ancestry). PRS were calculated using PRS‐CS‐auto with ancestry normalization (1000 Genomes European reference panel, HapMap3 SNPs, and excluding *APOE* region). Logistic regression assessed the association between each PRS and AD status, stratified by sex, adjusting for age, *APOE* status, and population stratification. Fisher’s Z test was used to compare the effect sizes between males and females.

**Result:**

In the overall cohort, PRS for education, AUDIT, pulse pressure, and Diastolic BP were significantly associated with reduced AD risk, while PRS physical activity, LDL and total cholesterol level were associated with increased AD risk. In sex stratified analyses, AUDIT, pulse pressure, and Diastolic BP showed association with reduced risk only in males. In contrast, LDL cholesterol, total cholesterol, and meat‐related diet PRS were associated with increased AD risk only in females. However, only the education PRS showed a significant sex difference in its effect (Fisher Z=‐2.57, *p* =0.010), with a stronger protective association observed in women (OR [95%CI]=0.87[0.85‐0.91], *p* =3.01e^‐15^) compared to men (OR [95% CI]=0.94 [0.90‐0.98], *p* =1.86e^‐3^).

**Conclusion:**

Our findings highlight that incorporating PRS into a sex‐stratified framework can uncover differential influences of modifiable risk factors on AD. Future work using Mendelian Randomization will evaluate whether these risk factors have a direct causal impact on AD development.